# BIOMARKERS IN CNS-ORIGINATING EXTRACELLULAR VESICLES: A DIAGNOSTIC META-ANALYSIS FOR PARKINSONIAN DISORDERS

**DOI:** 10.1093/geroni/igad104.3203

**Published:** 2023-12-21

**Authors:** Hash Brown Taha, Aleks Bogoniewski

**Affiliations:** University of Southern California, Los Angeles, California, United States; University of California Los Angeles, Los Angeles, California, United States

## Abstract

Parkinsonian disorders, including Parkinson’s disease (PD), multiple system atrophy (MSA), dementia with Lewy bodies (DLB), progressive supranuclear palsy (PSP), and corticobasal syndrome (CBS), present overlapping early symptoms, complicating accurate diagnosis. Predicting the conversion of prodromal conditions like REM behavior disorder (RBD) to PD, MSA, or DLB is also complex. Extracellular vesicles (EVs), small structures released by cells, carry cell-state-specific biomarkers that can cross the blood-brain barrier, providing a window into the brain’s biochemistry. Blood-isolated CNS-originating EVs have become a popular diagnostic tool. Yet, challenges remain in replicating and validating these findings. We conducted a PRISMA-guided systematic review and meta-analysis of 15 studies involving 1,455 persons with PD, 206 with MSA, 21 with DLB, 172 with PSP, 152 with CBS, 189 with RBD, and 1,045 healthy controls (HCs). Diagnostic accuracy for distinguishing persons with PD from HCs was moderate but showed high heterogeneity and significant publication bias. This indicates that studies with non-significant or lower effect sizes were less likely to be published. Differentiating persons with PD from those with PSP or CBS is limited due to the small number of involved studies. Our analyses suggest that biomarkers from CNS-originating EVs may not reliably differentiate persons with MSA or RBD from HCs due to variable accuracy and high heterogeneity. Our findings highlight the moderate diagnostic accuracy of EV biomarkers in differentiating Parkinsonian disorders, emphasizing substantial heterogeneity and significant publication bias. The need for larger, more standardized, and unbiased studies is underscored to validate the utility of EV biomarkers as diagnostic tools.

